# On the Problem of the Determination of Sex

**Published:** 1903-10

**Authors:** B. S. Schultze

**Affiliations:** Honorary Fellow of the British Gynecological Society; of the American Association of Obstetricians and Gynecologists


### SELECTIONS.

### On the Problem of the Determination of Sex.

By PROF. B. S. SCHULTZE, (Jena).

Honorary Fellow of the British Gynecological Society; of the American Association of Obstetricians and Gynecologists.

(*British Gynecological Journal.*)

THE Vienna embryologist, Schenk, started an idea that the sex of children to be borne by a woman might be determined by modifying her metabolism, but, so far as I am aware, his views have not been accepted in any quarter. The considerations underlying them had no sufficient scientific foundation, nor have they been confirmed by the results of the experiment.

Schenk, however, raised again the old, old question, why the development of an ovum should lead in one case to a male, and in another to a female offspring, and I have now before me two recent works upon it which lead to contradictory conclusions, and which, in my opinion, are inaccurate in the way the inferences made have been drawn. Doederlein[1] holds that the theory that the sex of the human ovum is determined before fertilisation cannot be upheld in the face of the fact, demonstrated by statistics, that men who are old in comparison with their wives beget a larger proportion of boys than agrees with the average number of males among all children born. Lenhosték[2] maintains that even in the ovary the ovum already has the character of sex, and denies that the husband has any influence upon the sex of the child.

In 1855 I expressed my opinion that all the conditions necessary for the development of either one sex or the other from an ovum were decided in the ovary, and wrote:[3] "All twins with a common chorion,.like all double monsters, are of one and the same sex; reported cases of the contrary always prove, when accurately investigated, incorrect, the apparent difference in sex turning out to be no more than an arrested development of the genital organs. When in connection with this we also consider the fact that actual hermaphrodism, the co-existence in one individual of testes and ovaries, of masculine and feminine germ-furnishing organs, has never been observed in the human being, nor, save perhaps in the most exceptional cases, in the other mammalia, it is evident that from a mammalia ovum only one sex, either masculine or feminine, can develop. Moreover, it is certain that, by the simultaneous fertilisation of more than one ovum, embryos of different sex may develop, and it is therefore probable not only that the cause of sex does not lie in the seed of the male, but rather that the condition for the development of the one or other sex are present in the ovum even in the ovary."

No one now maintains the old ideas that one ovary furnishes male, the other female ova; one testicle male and the other female seed; the evidence against them is too strong. Both boys and girls often enough have developed from the ova of one ovary after the extirpation of the other, and have been begotten by seed from one testicle after the removal of the other. Nor does anyone still believe that from an embryo, as long as it does not exhibit characters of its sex, that is to say, in the human being for about six or seven weeks, an infant of either sex may possibly develop.

Doederlein very justly says that it is not consistent with the view that an embryo already developing in the womb can be of undetermined sex to suppose that the father exerts any influence upon the sexual character of the offspring. Yet certain ascertained statistics offer very strong evidence of the action of such an influence. In the first place, the older the father is, in comparison with the mother, the more does the excess of male infants exceed the average proportion of male births (Hofacker, Sadler and others); and secondly, to breeders of horses and cattle it is a well-known fact that the stallion or bull upon whom more demands are made, which is allowed to cover sixty or even more females in the year, will beget a larger proportion of male offspring than one which has to fertilise only twenty or thirty females.

From the first of these facts Doederlein concludes that it is not right to suppose that the ovum is primitively endowed with a definite sex, "otherwise it is self-evident that the age of the begetter could not be a factor in the determination of the sex of the offspring." Of course it could not in that of the embryo from any particular ovum, but very well might be in determining the proportion of sexes born. It is quite possible that the seed of the older man is more adapted to fertilise male than female ova, and though we do not yet absolutely know that this is the case, I think it important to point out that the facts ascertained by Hofacker and Sadler are not conclusive proof that the ovum in the ovary is not of a definite sex.

Lenhossék argues in favor of the definite sex of the ovum in the ovary on the ground especially of thoroughly discussed biological analogies, as well as on that of the identical sex of uniovular twins. At the conclusion of his treatise he says: "Scientific research has, as we have explained . . . led us to accept as a fundamental fact almost indubitable, that in the animal kingdom the determination of sex is a prerogative of the maternal organisation, and precedes the fertilisation of the ovum." And on an earlier page: "Men must therefore resign themselves to the idea that to them no direct influence in the determination of the sex of their children is accorded, and that this determination is entirely left to the organism of the female individual." The possibility of even an indirect influence he only admits so far as, for example, the peculiarity of having more male children, may be transmitted through the son to the granddaughter. The ascertained statistics of Hofacker and Sadler, which he terms supposititions and hypotheses, he treats as controverted, though to me they seem to be facts as well ascertained as the above-mentioned results in horse and cattle breeding.

The apparent contradiction between these facts and the theory that the ovum in the ovary has a definite sex disappears if we suppose that the seed of the older man is better adapted to fertilise the male than the female ova of the younger woman; that spermatozoa fresh from the testicle of an actively-employed stud male is more effective in impregnating the male than the female ova of the dam.

Or the hypothesis may be put in this way. The male ova derived from the ovary of a young woman offer more attractions to the spermatozoa of an older man than the female. The male ova of the dam are more accessible to spermatozoa coming fresh from the testicle of the covering male than the female, and the latter are, on the whole, more accessible to spermatozoa which have for some time been ready awaiting their discharge from the male organs.

It is at all events, certain that even the prerogative of the male to influence the comparative number of his male and female offspring is not inconsistent with the theory that even in the ovary the sex of every ovum is already decided.

REFERENCES.

1. Doederlein: *"Urber Entstehung und willkurlich Bestimmung des Geschlechts," Deutsche Revue,* 1902.

2. v. Lenhossék: *"Das Problem der geschlechtsbestimmenden Ursachen."* Jena: Gustav Fischer, 1903.

3. Bernhard Schultze: *"Ueber anomale Duplicitaet der Anexorgane," Virchow's Archiv f. path. Anat.,* Bd. vii., p. 479.

